# Uptake, Growth, and Pigment Changes in *Lemna minor* L. Exposed to Environmental Concentrations of Cylindrospermopsin

**DOI:** 10.3390/toxins11110650

**Published:** 2019-11-07

**Authors:** Nelida Cecilia Flores-Rojas, Maranda Esterhuizen-Londt, Stephan Pflugmacher

**Affiliations:** 1 Institute of Ecology, Technische Universität Berlin, Ernst-Reuter-Platz 1, 10587 Berlin, Germany; nceci33@yahoo.com; 2 Faculty of Biological and Environmental Sciences, Ecosystems and Environmental Research Programme, University of Helsinki, Niemenkatu 73, 15140 Lahti, Finland; stephan.pflugmacher@helsinki.fi; 3 Korea Institute of Science and Technology Europe (KIST), Joint Laboratory of Applied Ecotoxicology, Campus 7.1, 66123 Saarbrücken, Germany; 4 Helsinki Institute of Sustainability Science (HELSUS), University of Helsinki, Fabianinkatu 33, 00014 Helsinki, Finland

**Keywords:** cylindrospermopsin, uptake, pigment contents, relative plant growth, *Lemna minor*

## Abstract

Cylindrospermopsin (CYN)-producing cyanobacterial blooms such as *Raphidiopsis*, *Aphanizomenon*, *Anabaena*, *Umezakia*, and *Lyngbya* spp. are occurring more commonly and frequently worldwide. CYN is an environmentally stable extracellular toxin, which inhibits protein synthesis, and, therefore, can potentially affect a wide variety of aquatic biota. Submerged and floating macrophytes, as primary producers in oligotrophic habitats, are at risk of exposure and information on the effects of CYN exposure at environmentally relevant concentrations is limited. In the present study, we investigated CYN uptake in the floating macrophyte *Lemna minor* with exposure to reported environmental concentrations. The effects were evaluated in terms of bioaccumulation, relative plant growth, and number of fronds per day. Variations in the concentrations and ratios of the chlorophylls as stress markers and carotenoids as markers of oxidative stress defense were measured. With exposure to 25 μg/L, *L. minor* could remove 43% of CYN within 24 h but CYN was not bioaccumulated. Generally, the pigment concentrations were elevated with exposure to 0.025, 0.25, and 2.5 μg/L CYN after 24 h, but normalized quickly thereafter. Changes in relative plant growth were observed with exposure to 0.25 and 2.5 μg/L CYN. Adverse effects were seen with these environmentally realistic concentrations within 24 h; however, *L. minor* successfully recovered within the next 48–96 h.

## 1. Introduction

Cylindrospermopsin (CYN) is an alkaloid with a tricyclic guanidine zwitterionic structure attached to a hydroxymethyluracil [1], which makes the toxin highly water-soluble. Two natural variants of CYN have been identified, namely 7-epicylindrospermopsin and deoxycylindrospermopsin [2,3]. Nowadays, CYN is recognised as a potent cyanobacterial toxin detected in waterbodies worldwide [4] and more often considered for its toxic consequences. The ever-increasing global occurrence of massive and prolonged blooms of cylindrospermopsin-producing cyanobacteria, such as *Raphidiopsis*, *Aphanizomenon*, *Anabaena*, *Umezakia*, and *Lyngbya* spp. poses a potential threat to both human health and the ecosystem balance [4,5]. Due to its predominantly extracellular availability because of active excretion, stability under a wide range of conditions, and ability to covalently bind to DNA/RNA [6] as well as to inhibit protein synthesis, CYN has the potential to impact a wide variety of aquatic plant and animal species [5,7]. Only a few studies examining the uptake of CYN have focused on aquatic plants and investigated the subsequent physiological responses and effects on growth associated with CYN exposure. Submerged and emerged macrophytes, as the primary producers in oligotrophic habitats, play an important role in stabilising aquatic ecosystems, where the consumers in all trophic levels depend on them. They are vital in the aquatic carbon cycle, also providing shelter for young animals, egg-hatching niches and shade to cool the surface waters [8]. Their central role thus emphasises the need to understand how they are affected by hazards in their environment.

Studies focusing on CYN assimilation in aquatic plants have shown low uptake concentrations and associated bioconcentration factors (BCF). White et al. [9] reported a maximum uptake of 15 ng CYN/g FW in *Hydrilla verticillata* (BFC 0.045) after seven days with exposure to 400 μg/L CYN. Interestingly, exposure for longer periods (up to 14 days) did not result in bioaccumulation. In *Landoltia punctata* (previously *Spirodella oligorrhyza),* exposed to *Cylindrospermopsis raciborskii* whole-cell extracts (containing 0 to 500 μg/L CYN), 30 ng CYN/g FW plant material was internalised (BFC 0.038) after six days of exposure to the highest CYN concentration [10]. Santos et al. [11] exposed *Azolla filiculoides* to crude extracts with three different CYN concentrations (50, 500, and 5000 μg/L). After seven days of exposure, CYN uptake (1.31 ± 0.11 µg/g plant material fresh weight (FW); BCF 0.40 ± 0.04) could only be quantifiable in plants exposed to the highest CYN concentration.

The aquatic fern *A. filiculoides* showed a 99.8% growth inhibition after seven days of exposure to 5000 μg/L CYN; although, not with lower exposure concentrations [11]. The same study reported increased chlorophyll, carotenoid, and protein contents. Jambrik et al. [12] studied the growth alterations in *Lemna minor* and *Wolffia arrhiza* exposed to a crude extract containing CYN as well as the purified toxin (ranging from 10 to 20,000 μg/L). Both crude extract and pure CYN induced growth alterations, such as decreased frond numbers and fresh weight in both plants after five days of treatment. Several studies reported the stimulation of root growth and reduced leaf fresh weight with exposure to CYN either as a purified toxin or in a crude extract in plants such as *H. verticillata* [13] or *Oryza sativa* [14].

CYN exposure has been associated with instigating oxidative stress in plants, as shown in *O. sativa* [14], and *A. filiculoides* [11]. Few studies investigating the effect of CYN have been conducted with duckweed species. *L. minor* exposed to CYN showed increased catalase (CAT) activity within 168 h of exposure to CYN concentrations of 2.5 and 25 μg/L, respectively [15]. In the same study, glutathione S-transferase (GST) and glutathione reductase (GR) activities increased after 24 h with 25 μg/L CYN. However, the highest activities were recorded after 168 h only at the highest CYN concentration [15]. The authors suggested that CAT plays a vital role as a first mechanism to control oxidative stress, while GST and GR play a crucial role during longer exposure times and at high CYN concentrations.

Although research regarding the effects of CYN in aquatic environments has increased in the last years, the uptake, corresponding effects on growth, and the physiological changes in macrophytes at environmentally relevant concentrations have not yet been studied extensively. Due to the pivotal role of macrophytes in the aquatic environment, understanding these effects is required. The information is moreover necessary to understand how CYN is metabolized and the effects on the plant defence mechanisms, to further assess the applicability of these plants in phytoremediation technologies for cyanobacterial toxins such as the Green Liver System^®^ [16].

The aquatic macrophyte *L. minor*, commonly known as duckweed, is widely used in aquatic testing [17] and has been reported to be a potential scavenger of heavy metals from aquatic environments and are being applied in wastewater treatment and phytoremediation [18]. To further investigate the application range of *L. minor* in phytoremediation, the present study investigated its uptake ability and the corresponding physiological responses with exposure to pure CYN. The pigment contents such as chlorophyll *a* and *b* and carotenoids, as well as the number of fronds, were used as indicators of changes in the plant’s stress status in response to CYN exposure and uptake. On this basis, the study assessed the connection between the physiological responses in this macrophyte and if it can be used for phytoremediation as the inability of an aquatic plant to cope with toxin exposure on a physiological level may lead to death and re-release of xenobiotics already taken up. Furthermore, CYN studies to date have been conducted with concentrations far higher than those detected in the environment [4,19,20]; therefore, in the current study, concentrations resembling environmental scenarios were used.

## 2. Results and Discussion

### 2.1. CYN Uptake

**Figure 1:**
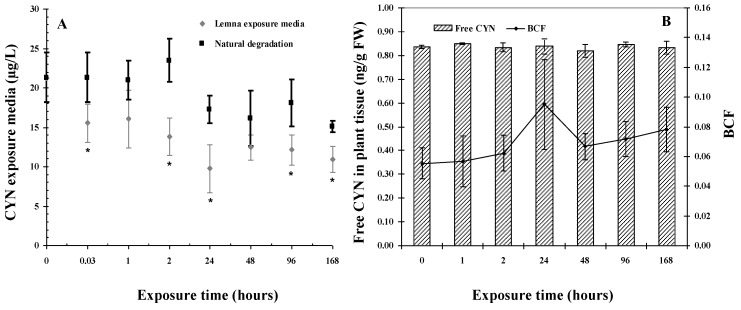
(**A**) CYN concentration in the exposure media in the presence (treatment) and absence (positive control) of *L. minor* with time (**B**) CYN concentration taken up by *L. minor* with time and corresponding BCFs. Data represent the mean value ± standard deviation (*n* = 4). Significances compared to the media control are indicated by the asterisks (* *p* < 0.05).

In the negative control plants, no CYN contamination was detected. For the positive control (Figure 1A), 19% of the CYN in the media was degraded after 24 h, which further increased to 29% after 168 h. The low natural degradation of CYN previously proven [7,21,22,23], was thereby confirmed in the present study. The degradation was assumed to have occurred due to light and temperature effects or bacterial contamination or possibly protein binding [24].

The CYN concentrations in the exposure media containing plants were significantly reduced compared to the positive control, which lacked plants (*p* < 0.05) within a minute after beginning the experiment (0.03 h; Figure 1A). At this time, the CYN concentration in the media containing plants decreased from 21.29 ± 3.1 µg/L to 15.55 ± 2.41 µg/L corresponding to 27% CYN removed. Natural degradation is not likely to occur this fast [7]; therefore, it is assumed that *L. minor* was responsible for the removal. The media CYN concentration in the treatments decreased sharply within the first 24 h to 9.76 ± 3.04 μg/L (*p* < 0.005), corresponding to a removal percentage of 54%. Taking into account natural degradation, the percentage of CYN removed by *L. minor* after 24 h was 43%. After 96 h and 168 h, the CYN concentrations in the exposure media were significantly lower compared to the control (*p* < 0.001). However, at 48 h, 96 h, and 168 h, the CYN removal percentages remained similar among them (*p* > 0.05). The highest rate of CYN removal corresponds to 861 ng/min (0.03 h), followed by 13.08 ng/min and 9.29 ng/min after 1 h and 2 h, respectively, while after 24 h, no further uptake was documented. The results suggest that *L. minor* could take up CYN most effectively during the first periods of exposure, which is not only consistent with previous studies on CYN uptake in macrophytes [9], but also other cyanotoxins [25,26,27,28].

Free CYN was detected in the plant tissues for each exposure time ranging from 0.82 ± 0.03 ng/g FW to 0.85 ± 0.01 ng/g FW (Figure 1B). Free CYN in the plant tissues, and thus the BCFs, did not show significant changes between the different exposure times (*p* > 0.05); therefore, metabolism or biotransformation of CYN is unlikely. The highest bioconcentration factor (BCF) was 0.095 ± 0.03, which was achieved after 24 h of exposure (Figure 1B). The low amounts of free CYN in the tissues and no bioaccumulation (BCF < 1) reported in this study are similar to previous studies related to CYN exposure at higher concentrations [9,10,11]. In the current study, the rapid partial removal of CYN within 2 h could be, in part, attributed to the adhesion of CYN on the cell wall because of its zwitterionic properties together with microbial degradation. White et al. [9] discussed the absence of free-CYN bioconcentration in *H. verticillata*, leaving open the question of whether CYN can become intracellular but is transported out of the cell at the same rate at which it enters; or that intracellular toxin could be enzymatically bound, modified or metabolized within plants preventing detection with quantitative techniques used. A recent study with crude lysates from different aquatic organisms exposed to 25 μg/L CYN has shown a significant decrease in free CYN percentages. The same study also revealed that the preparation of the proteins influences the percentage of free CYN detectable and that all CYN could be accounted for when incubated with amino acids and oxidised or reduced glutathione, suggesting protein binding [24].

*E. densa* exposed to 50 μg/L CYN displayed 0.05% toxin uptake per exposure organism [29]. In the present study, considering the CYN remaining from the media after the exposures with *L. minor*, free CYN in the whole exposed plant biomass represented between 0.15% to 0.30% of CYN removed in water. The low percentages of CYN in plants suggest that cyanotoxin could be binding to components of the plant cells. The previous investigations and the current study cannot confirm the association of CYN to proteins but highlights the need to continue investigating this subject.

### 2.2. Photosynthetic Pigment Contents

**Figure 2:**
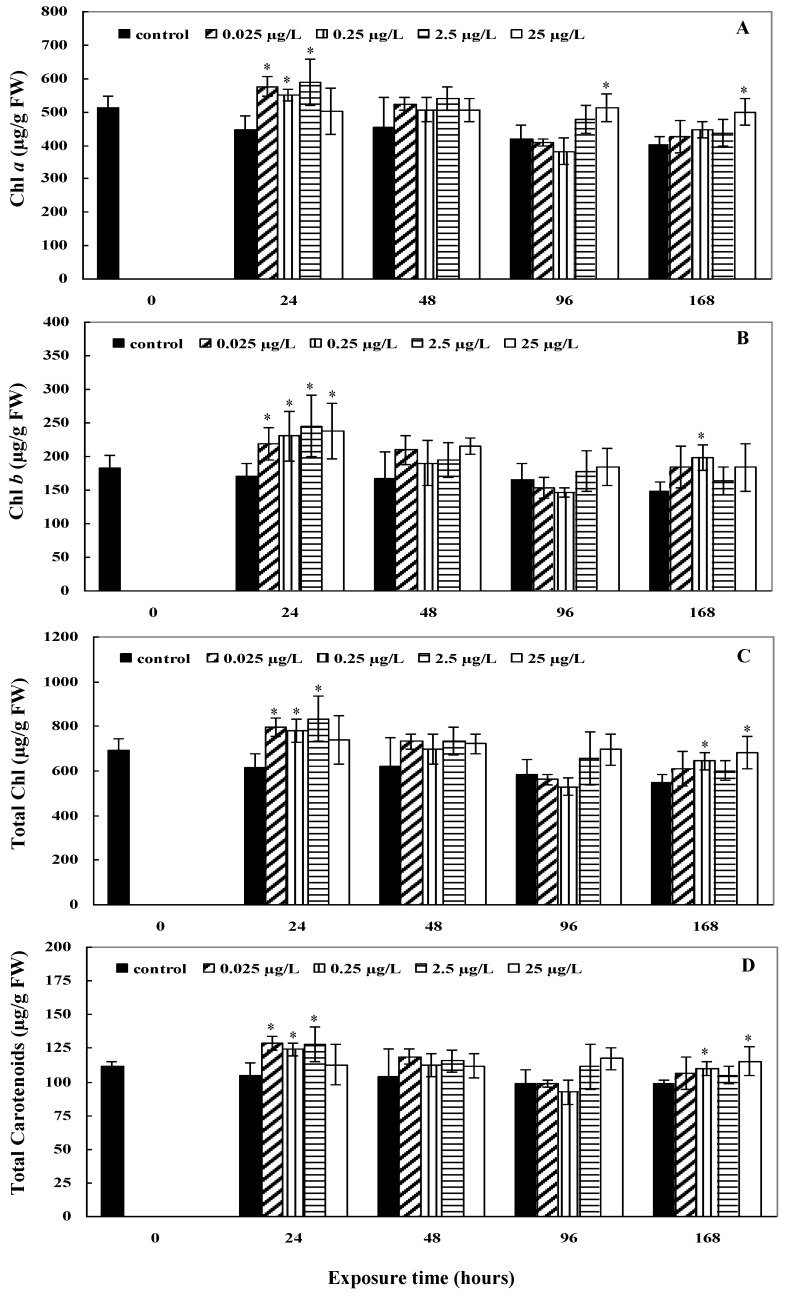
The (**A**) chlorophyll *a*, (**B**) chlorophyll *b*, (**C**) total chlorophyll, and (**D**) carotenoid content in *L. minor* with exposure to different concentrations of CYN. Data represent mean pigment concentration ± standard deviation of three independent samples, each determined three times (*n* = 9). Significance compared to the control is shown by an asterisk (* *p* < 0.05).

The chlorophyll *a* content in *L. minor* exposed to CYN increased relative to the control after 24 h with all exposure concentrations (*p* < 0.05) except 25 μg/L (Figure 2A); however, this returned to the same concentration as the control within 48 h (*p* > 0.05). With exposure to 25 μg/L, a later (at 96 h) but prolonged elicitation until 168 h was seen (*p* < 0.05). The chlorophyll *b* and total chlorophyll contents reacted similarly with a sharp increase after 24 h of exposure, followed by normalisation after 48 h (Figure 2B,C). Enhanced carotenoid content compared to the control was observed after 24 h with 0.025 μg/L, 0.25 μg/L, and 2.5 μg/L CYN exposure concentrations (*p* < 0.05). After 168 h, the carotenoid concentration remained significantly increased compared to the control for treatments with 0.25 μg/L and 25 μg/L CYN (*p* < 0.05) (Figure 2D).

**Figure 3:**
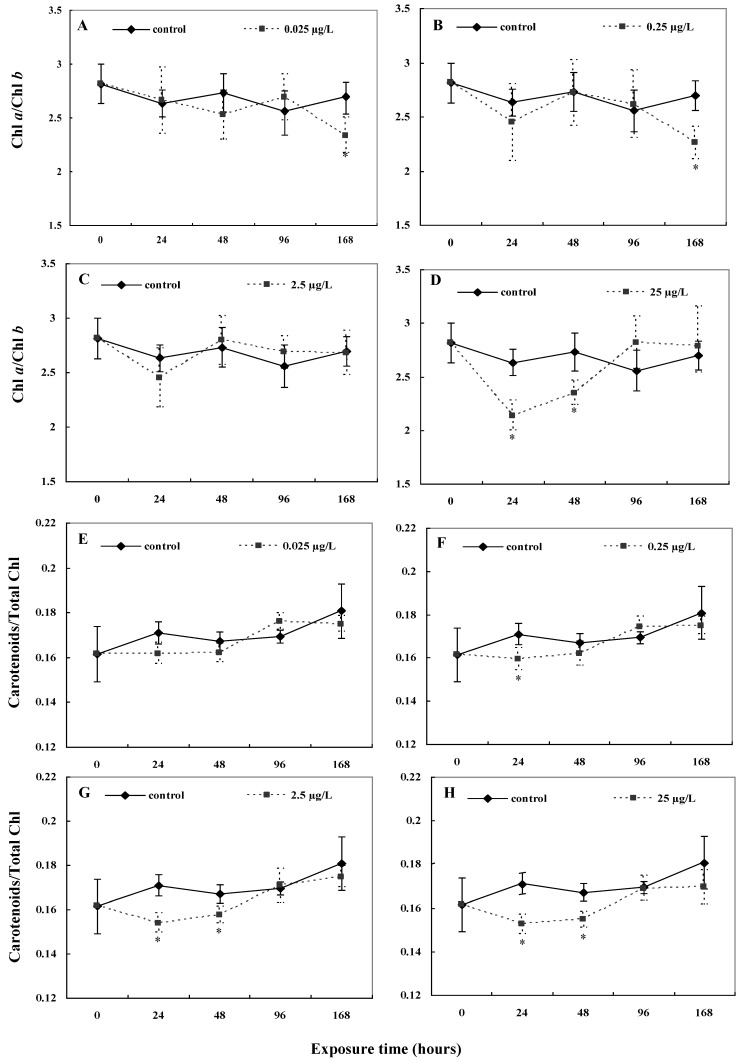
The ratio of chlorophyll *a* to *b* in *L. minor* with exposure to (**A**) 0.025 μg/L, (*B*) 0.25 μg/L, (**C**) 2.5 μg/L, and (**D**) 25 μg/L CYN as well as the ratio of carotenoids to total chlorophyll with exposure to (**E**) 0.025 μg/L, (**F**) 0.25 μg/L, (**G**) 2.5 μg/L, and (**H**) 25 μg/L CYN. Significance compared to the control is shown by an asterisk (* *p* < 0.05).

The chlorophyll *a* to chlorophyll *b* ratio showed different alterations concerning the four CYN concentrations used (Figure 3A–D). With exposure to 0.025 μg/L and 0.25 μg/L CYN, a significant decrease in the chlorophyll *a* to *b* ratio was only seen after 168 h (*p* < 0.05) (Figure 3A,B). No significant effects were observed with exposure to 2.5 μg/L (Figure 3C). After 24 h, the chlorophyll *a* to *b* ratio decreased with exposure to 25 μg/L CYN only (*p* < 0.05), which then returned to the statistically similar ratio as the control (*p* > 0.05) (Figure 3D). The carotenoids to total chlorophyll ratio decreased significantly compared to the control after 24 h with exposure to 0.25 μg/L, 2.5 μg/L, and 25 μg/L (*p* < 0.05; Figure 3F–H), but recovered within 48 to 96 h of the disturbance. With exposure to 0.025 μg/L, no significant alterations in the ratio of carotenoids to chlorophyll were observed (Figure 3E). 

The results suggest that exposure to CYN causes an increase in the concentration of the chlorophyll *a* and carotenoids within 24 h at CYN concentrations under 25 µg/L. In previous studies, the effects reported on the chlorophyll *a* and *b* contents with exposure to CYN have been contradictory [11,13]. Increased chlorophyll *a* content, accompanied by increased carotenoids, may indicate an effect on the maintenance of the complex chlorophyll-carotenoid binding proteins, which are responsible for the absorption and conversion of light energy during photosynthesis. The increase of the carotenoids after 24 h suggest their activity in response to exposure to the xenobiotic CYN in the antioxidative system to combat excessive ROS formation [30]. For the highest CYN exposure concentration (25 μg/L), the stabilisation could only occur later (96 to 168 h), probably after initial damage due to exposure was overcome by the antioxidative stress enzymes [15]. Santos et al. [11] also reported increased carotenoid levels with CYN exposure in *A. filiculoides*. The changes in the carotenoid contents may be related to ROS production and plant growth. ROS production and signalling are integrated with the action of phytohormones in the coordinate regulation of plant growth and stress tolerance [31]. Many plant hormones generate ROS as part of the mechanism that regulates plant growth and development [31]. Moreover, the observed changes in the carotenoids to total chlorophyll ratios showed the stabilisation of the photosynthetic system in *L. minor* after 96 h for the higher CYN concentrations.

### 2.3. Plant Growth

**Figure 4:**
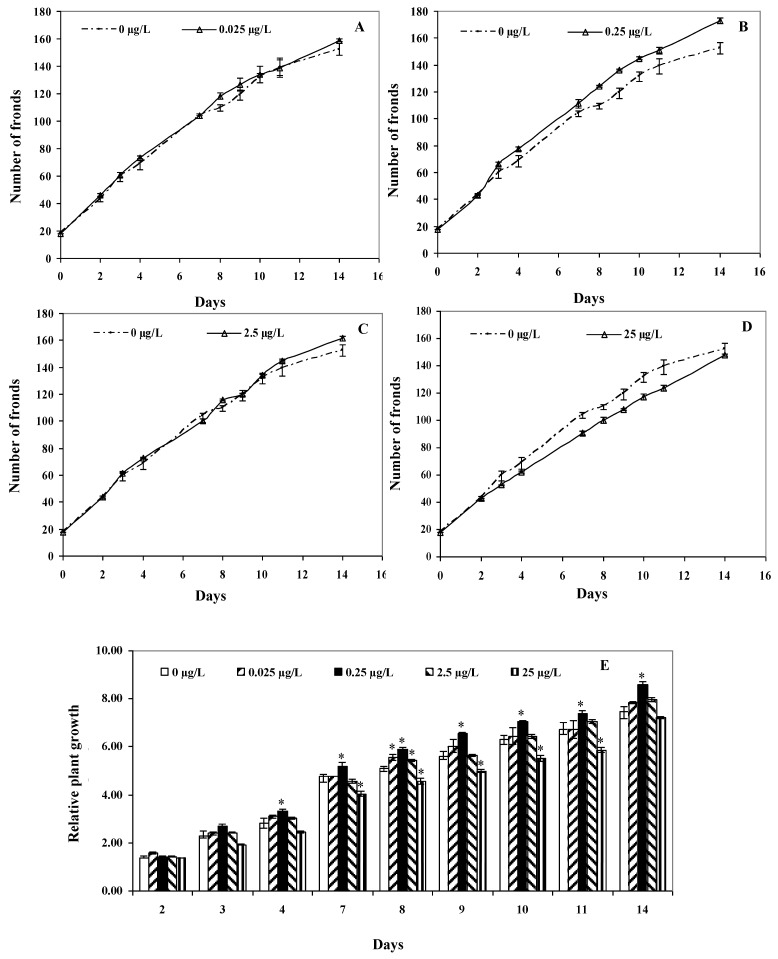
The growth of *L. minor* in terms of mean number of fronds (*n* = 3) with exposure to (**A**) 0.025 μg/L, (**B**) 0.25 μg/L, (**C**) 2.5 μg/L, and (**D**) 25 μg/L CYN, as well as mean relative plant growth with exposure to the four CYN concentrations (**E**). Significance compared to the control is shown by an asterisk (* *p* < 0.05).

In general, changes in the number of fronds with exposure to the different CYN concentrations were divergent. The number of fronds with exposure to 0.25 and 2.5 μg/L CYN were higher compared to the control (Figure 4B,C), while for 25 μg/L CYN, the number of fronds decreased relative to the control (Figure 4D). With exposure to the lowest concentration, 0.025 μg/L CYN, no effects were observed (Figure 4A).

The *L. minor* growth evaluated by calculating the relative plant growth (RG) aswell was dissimilar for the four CYN treatments (Figure 4E). After 4 days, the RG increased significantly only with exposure to 0.25 μg/L CYN (*p* < 0.05) and remained significantly higher until the end of the exposure period. Interestingly, after 7 days, the RG of the plants grown in the presence of 25 μg/L decreased significantly compared to the control (*p* < 0.05). After the 8th day of exposure, the RG for plants exposed to 0.025 μg/L and 2.5 μg/L showed a significant increase. Except for plants exposed to 0.25 μg/L and 25 µg/L CYN, the RG recovered by the next sampling point.

In two studies by Kinnear et al. [10,13] with *L. punctata* and *H. verticillata*, an increased relative growth was reported with exposure to CYN. Similarly, Santos et al. [11] reported an increase in the growth of *A. filiculoides* with exposure to CYN and attributed the observation to it being an approach to combat the toxicity.

The higher increase in the number of fronds at lower concentrations can be related to hormetic responses, where low-stress conditions can induce growth. Hormesis has been demonstrated in *L. minor* exposed to different pesticides [32]. However, this study cannot confirm the hormetic response in *L. minor* exposed to CYN, but this assumption could be considered in other investigations.

The decreases of the chlorophyll *a* to *b* ratio seen at the low exposure concentrations after 168 h may be explained by the higher increases in the number of fronds at these concentrations. The appearance of new fronds in *L. minor* occurs by division from mother fronds. The senescence of the mother fronds was observed in the experiment; this can affect the change of pigment contents. Additionally, more fronds at the same surface tend to compete for light.

The increase in the number of fronds below the control at the highest CYN concentration can be related to changes in the carotenoids to total chlorophyll ratios. Some phytohormones regulating growth derivate from carotenoid precursors [33]. A decrease in the carotenoid contents related to total chlorophyll can collaborate to delay growth. In this way, *L. minor* can reduce ROS formation from plant growth until the enzymatic antioxidant and non-enzymatic antioxidant system regulates oxidative stress caused by CYN. The stabilisation of the carotenoids to total chlorophyll ratio after 96 h and 168 h may show the regulation of biosynthesis and storage of carotenoids in *L. minor*, which, therefore, leads to stabilise growth.

## 3. Conclusions

In agreement with previous studies in aquatic macrophytes with high concentrations, CYN exposure at environmentally relevant concentrations did not result in bioaccumulation. In response to exposure, the pigment concentrations were elevated for a short period, probably to combat the adverse effects of the xenobiotic. Relative plant growth was stimulated; however, this was previously proposed to be a response to stress due to CYN exposure. *L. minor* can tolerate and even combat adverse effects experienced from exposure to CYN at environmentally reported concentrations; however, as it does not remove large amounts of CYN from its environment after the first 24 h of exposure or bioaccumulate CYN continuously, it is not an ideal candidate alone for sustainable phytoremediation. It is necessary to continue studies on CYN uptake and CYN removal with other macrophytes to be able to have more tools for designing methods to control CYN in aquatic environments.

## 4. Materials and Methods

### 4.1. Plant Material

*L. minor* was provided by Wakus (Wakus GmbH Wasserpflanzenkulturen, Germany). The identity of the macrophyte was verified according to Rothmaler [34] and in accordance with the rules given by the Botanical Society of Britain and Ireland [35]. A culture of *L. minor* was maintained in glass tanks (60 cm × 60 cm × 60 cm) in modified Provasoli’s medium consisting of de-ionized water containing CaCl_2_ (0.20 g/L), NaHCO_3_ (0.11 g/L), and sea salt (chloride, sodium, sulfate, potassium, calcium, carbonate, boron, magnesium, strontium; 0.10 g/L), under cool white fluorescent light (50 µE/m²·s irradiation measured with a light meter) with a 14:10 h light:dark photoperiod at 20 ± 1 °C [15]. These cultivation conditions were also used for all exposures.

### 4.2. Chemicals

CYN standard (*p*urify > 95%) was purchased from Alexis Biochemicals (Lausen, Switzerland) and dissolved in 70% methanol to obtain a stock solution. All chemicals used in Laboratory experiments were of analytical-grade quality and were obtained from Sigma-Aldrich, Inc. (Darmstadt, Germany).

### 4.3. CYN Treatments

Four concentrations of CYN (0.025, 0.25, 2.5, and 25 μg/L) were prepared from the stock by dilution with culture medium. The exposure concentrations were selected according to typical CYN concentrations previously recorded in aquatic environments [4,19,20]. Before the beginning of the experiment, the plants were pre-cultured in the exposure vessels to acclimate for seven days. The experiments were carried out with non-axenic plants under nonsterile conditions. Per treatment, fronds of *L. minor* with a total fresh weight (FW) of 2.5 ± 0.5 g were exposed to the four specified CYN concentrations, respectively, in a volume of 150 mL (surface area of 70.8 cm^2^) under the controlled culture conditions described above for 168 h. Each treatment and its negative control in parallel was prepared with its independent replicates.

For CYN uptake, only the highest exposure concentration of 25 μg/L CYN was used due to the limitations of the analytical method (limit of quantification was 10 pg on column; see Section 4.4). The treatment and control replicates (*n* = 4) were sampled after 0.02 h, 1 h, 2 h, 24 h, 48 h, 96 h, and 168 h of exposure in order to analyse the uptake of CYN using liquid chromatography–tandem mass spectrometry (LC–MS/MS, Section 4.4). Assuming that natural degradation of CYN could occur, an additional positive control (25 μg/L CYN medium without plants; *n* = 4) was set up concomitantly and sampled at the same exposure times.

The experimental setup was repeated for the analysis of the pigment content and plant growth with all four exposure concentrations (*n* = 3). Photosynthetic pigment contents were measured at all concentrations from the samples after 24 h, 48 h, 96 h, and 168 h and analysed as described in Section 4.5. After exposure, plants were washed twice with 100 mL of de-ionised water to remove any remaining toxin from the plant surfaces. The plants were shock frozen in liquid nitrogen and stored at −80 °C for further biochemical analysis.

### 4.4. Analysis of CYN

Free CYN was extracted from the plant tissue, according to Esterhuizen-Londt et al. [29], with slight modifications. The frozen plant material was ground in liquid nitrogen to a fine powder and lyophilised (LIO-5P freeze-dryer Kambič Laboratorijska oprema doo, Semič, Slovenija) overnight (−50.3 °C; 6.1 mbar). The lyophilised samples (500 mg) were further homogenised mechanically using a Tissuelyser LT (Qiagen, Hilden, Germany), then suspended in 200 µL 95% acetonitrile (ACN) followed by ultrasonic treatment for 30 min in an ultrasonic water bath (Alpax, Gmbh and Co. KG, Goldach, Switzerland). The samples were continuously shaken in the dark for 30 min at room temperature using an overhead Intelli-Mixer RM-2 (Neolab, Heidelberg, Germany). Extracts were then centrifuged at 20,800× *g* for 15 min at 4 °C (Eppendorf Centrifuge 5417 R, Hamburg, Germany). The pellets were suspended in 300 µL of 95% ACN and again, centrifuged as before. Supernatants were combined and analysed by LC–MS/MS. The concentrations of CYN in the exposure media were also analysed by LC–MS/MS.

Chromatographic separation of CYN was achieved with a Kinetex HILIC column (2.6 µm, 2.1 × 100 mm) by liquid chromatography (1200 infinity Series, Agilent, Waldbronn, Germany) coupled to triple quadrupole mass spectrometry (model 6460 Triple Q^TM^, Agilent) with electrospray ionization (Jet-Stream, Agilent, Santa Clara, CA, USA) according to Esterhuizen-Londt et al. [29]. The column oven temperature was set to 35 °C, and an injection volume of 10 µL was used for each sample at a flow rate of 0.5 mL/min. Compound separation was achieved using a gradient elusion starting at 95% ACN (MS grade) for 3 min, which was then decreased to 50% over 4 min with a post time of 3 min, resulting in a retention time of 4.1 min for CYN. For the subsequent MS–MS detection, the MRM mode (positive mode) was used with a mass transfer of 416 (Q1) to 176 and 194 (Q3) for CYN. The drying gas temperature and flow settings were 320 °C and 12 L/min, respectively and the sheath gas temperature and flow were set to 380 °C and 12 L/min using nitrogen gas. The capillary voltage applied was 4500 V, and the nozzle voltage was set to 1200 V, together with a nebuliser pressure of 25 psi. The calibrations used for quantification of CYN were linear (R^2^ = 0.998) between 0.01 and 100 μg/L. The limit of quantification was set at 10 pg on column (S/N ≥ 5).

The bioconcentration factors (BCF) per exposure time were calculated by dividing the CYN concentration in the plant tissue (ng/g FW) by the corresponding CYN concentration in the media (ng/mL) [36].

### 4.5. Photosynthetic Pigment Contents

Chlorophyll and carotenoid contents were measured according to Inskeep and Bloom [37] and Wellburn [38], respectively. The plant samples were ground to a fine powder in liquid nitrogen, and a total of 0.05 g FW were suspended in 5 mL of N,N-dimethylformamide (N,N-DMF) in the dark at 4 °C for three days. The extraction solution was centrifuged (Eppendorf Centrifuge 5417 R, Hamburg, Germany) at 20,800× *g* for 15 min at 4 °C. Spectrophotometric analysis was carried out in the dark in triplicate at 647 nm, 664.5 nm (for chlorophyll *a* and *b* respectively), and 480 nm (for carotenoids) with 1 cm quartz cuvettes.

### 4.6. Plant Growth Determination

*L. minor* plants were exposed to four concentrations of CYN (0.025, 0.25, 2.5, and 25 μg/L) for 14 days. The exponential growth period was previously assessed (doubling time of frond number <2.5 days). A total of nine individual colonies, each with two fronds, were inoculated in 100 mL CYN solution. The number of fronds from each treatment were counted from the second day and within two weeks. Plant growth was expressed as relative plant growth (RG) according to Equation 1 [39] as follows:

RG = (N_t_ − N_0_)/N_0_

where N_t_ is the number of fronds at day t and N_0_ is the number of fronds at the beginning of the experiment.

### 4.7. Statistics

All statistical analysis was performed using SPSS software (IBM SPSS Statistics, Version 20, IBM Corporation, New York, NY, USA). Differences between treatments (toxin concentrations) and corresponding controls were analyzed by one-way analysis of variance (ANOVA) followed by Tukey’s post-hoc at an alpha level of *p* = 0.05. Before the ANOVA test, normality and homogeneity of variance among groups were tested by Shapiro–Wilk and Levene’s test, respectively. When necessary, data were transformed for normalisation to reduce heterogeneity. When the assumptions of homogeneity were not satisfied, a nonparametric Levene’s test was performed. In this case, significant differences between treatments and controls were analyzed by the nonparametric Kruskal–Wallis test.
